# Clinical assessment and train-of-four measurements in critically ill patients treated with recommended doses of cisatracurium or atracurium for neuromuscular blockade: a prospective descriptive study

**DOI:** 10.1186/s13613-017-0234-0

**Published:** 2017-01-19

**Authors:** Pierre Bouju, Jean-Marc Tadié, Nicolas Barbarot, Julien Letheulle, Fabrice Uhel, Pierre Fillatre, Guillaume Grillet, Angélique Goepp, Yves Le Tulzo, Arnaud Gacouin

**Affiliations:** 1 Service des Maladies Infectieuses et Réanimation Médicale, CHU Rennes, 2 rue Henri Le Guilloux, 35033 Rennes, France; 2 Faculté de Médecine, Biosit, Université Rennes 1, 35043 Rennes, France; 3 Réanimation polyvalente, CH Saint-Brieuc, 22000 Saint-Brieuc, France; 4 Inserm-CIC-1414, Université Rennes I, IFRI 40, 35033 Rennes, France

**Keywords:** Intensive care unit, Muscular weakness, Neuromuscular blockade, Train-of-four monitoring, Benzylisoquinoliniums

## Abstract

**Background:**

Despite few studies, a monitoring of a neuromuscular blockade with a train of four (TOF) is recommended in intensive care unit (ICU). Our objective was to compare the results of ulnar and facial TOF measurements with an overall clinical assessment for neuromuscular blockade in ICU patients treated with recommended doses of atracurium or cisatracurium, including patients with acute respiratory disease syndrome (ARDS).

**Methods:**

We prospectively included in two ICUs 119 patients, 94 with ARDS, who required a neuromuscular blockade for more than 24 h. Three levels of neuromuscular blockade were defined: “over-paralyzed” (TOF = 0), “well-paralyzed” (TOF = 1–2), and “under-paralyzed” (TOF = 3–4). Physicians blinded for TOF counts were asked to classify patients clinically as “over-paralyzed,” “well-paralyzed,” or “under-paralyzed”. Patients were assessed two times daily.

**Results:**

For the whole population 996 ulnar and facial TOF measurements and clinical assessments were obtained (846 with cisatracurium and 150 with atracurium). Proportions of patients classified as over-paralyzed, well-paralyzed, and under-paralyzed based on TOF measurements and clinical assessments differed significantly (*p* < 0.0001). The number of observed agreements between clinical assessments and facial TOF measurements was of 19.08% (*κ* = 0.06) and of 17.37% with ulnar TOF measurements (*κ* = 0.04), while it was of 62.75% between ulnar and facial TOF measurements (*κ* = 0.45). Results were similar for cisatracurium and atracurium. Repeated facial TOF measurements performed on the first 4 days of mechanical ventilation in ARDS patients showed that the proportion of patients TOF = 1–2 was around 8% and did not vary significantly with time (*p* = 0.9), proportion of patients TOF = 3–4 increased from 24 to 40% (*p* = 0.01), proportion of patients TOF = 0 decreased from 71 to 53% (*p* = 0.005) while objectives for protective ventilation were achieved. Proportions of facial and ulnar TOF = 0 were significantly higher among patients with ICU-acquired weakness (ICU-AW) versus those who did not develop ICU-AW (51 vs. 40%, *p* = 0.03, and 76 vs. 62%, *p* = 0.006, respectively).

**Conclusions:**

The study provides data on clinical and TOF monitoring of neuromuscular blockade, which are widely divergent in ICU patients receiving recommended doses of benzylisoquinoliniums.

**Electronic supplementary material:**

The online version of this article (doi:10.1186/s13613-017-0234-0) contains supplementary material, which is available to authorized users.

## Background

Neuromuscular blocking agents (NMBAs) are often used in association with adequate analgesia and sedation for the following conditions: management and facilitation of mechanical ventilation (MV), management of elevated intracranial or intraabdominal pressure, treatment of muscle spasms, and reduction in oxygen consumption [1, 2]. Recently, NMBAs became common intravenous medications used within the intensive care unit (ICU) since Papazian et al. [3] enhanced the role of NMBAs when they found a beneficial effect of a neuromuscular blockade on the mortality in acute respiratory distress syndrome (ARDS). The previous recommendations of the American and French critical care societies for sustained neuromuscular blockade are of C grade for indications and of B grade for monitoring [2, 4]. In the clinical practice guidelines for sustained neuromuscular blockade in the adult critically ill patient published in 2016 [5], NMBAs are suggested in ARDS patients with a ratio PaO2/FiO2 less than 150 (weak recommendation) but no dosage is mentioned. All these recommendations [2, 4, 5] suggest that in ICU patients the appropriate depth of neuromuscular block may be variable and depends on the reason for neuromuscular blockade, the expected patient outcome, and the phase of the disease. Monitoring the depth of a neuromuscular blockade with peripheral nerve stimulation (PNS) is recommended only in combination with a clinical assessment by critical care societies [2, 4, 5]. The most commonly used tool is the train of four (TOF), and previous recommendations suggest dosing titration of NMBAs to one or two visualized muscle twitches [2, 4]. Of note, in the study of Papazian et al. [3], PNS was not permitted and in the 2016 practice guidelines [5] no TOF objective is recommended. North American surveys performed in the 2000s reported that 84–91% of physicians used PNS for monitoring but also that neuromuscular blockade practices varied greatly between intensive care units (ICUs) [6, 7]. Using TOF monitoring in the ICU may result in a reduction of NMBAs’ dose and a subsequent decrease risk of complications related to prolonged and/or excessive blockade, such as intensive care unit-acquired weakness (ICU-AW) [8, 9]. The benefits and limitations of TOF stimulation were primarily determined from studies performed in operative room patients [10, 11], with results that may not be generalizable to ICU patients. In critically ill patients, the duration of neuromuscular blockade is longer, sepsis and/or shock is often present, and pharmacokinetic is difficult to predict [12–14]. Lastly, agreement between subjective and objective means of assessing the degree of neuromuscular blockade has been little studied in ICU [15–17]. Thus, we designed a prospective observational study in two ICUs using NMBAs with infusion rates following recommendations with the aims (1) to describe and compare TOF measurements obtained at ulnar and facial sites with clinical evaluation of depth blockade, (2) to focus on the results obtained in ARDS patients with time analysis, and (3) to evaluate when available muscle strength 7 days after awakening the patient.

## Methods

### Patients and setting

The study was conducted between April 1, 2014, and March 31, 2015, in two different ICUs: in a 21-bed medical ICU in Rennes University Hospital (center 1) and in a 14-bed mixed medical-surgical ICU in Saint-Brieuc General Hospital (center 2). The study was approved by the Rennes University Hospital’s ethic committee (No. 14-17), and no informed consent was required because of the observational nature of the study. A non-opposition form was provided to families.

All patients aged over 18 years with a planned duration of a neuromuscular blockade for more than 24 h were screened, and those treated at least 24 h with NMBAs were finally included for analysis. Exclusion criteria were contraindications to NMBAs (malignant hyperthermia, history of anaphylaxis with NMBAs), the inability to assess muscle strength or TOF count (neuromuscular disease, admission for cardiac arrest, body core temperature below 36.5 °C), pregnancy, moribund state, decision to withhold life-sustaining treatment, refusal of the family, and continuous infusion of NMBAs for more than 12 h before screening.

### Neuromuscular blockade

Before neuromuscular blockade, sedatives and opioids were titrated by nurses according to the Ramsay Sedation Scale in center 1 [18] with an objective of 6 and the Richmond Agitation Sedation Scale in center 2 [19] with an objective of −5. There was no daily sedation interruption. Sedatives used were midazolam or propofol, and opioids used were sufentanil or morphine. The NMBAs used were benzylisoquinoliniums: cisatracurium in center 1 and atracurium in center 2. Boluses and initial infusion rates followed the 2002 and 2008 recommendations using patient’s body weight on admission: 0.15 ± 0.05 and 0.5 ± 0.2 mg kg^−1^ h^−1^ for cisatracurium and atracurium, respectively [2, 4]. The management of the neuromuscular blockade was left to the physician in charge, who was blinded about results of the TOF, according to its clinical evaluation. Total doses, durations of treatment, and NMBAs’ infusion rates were recorded daily.

The PNS used were TOF-Watch-S^®^ (Organon Ltd., Dublin, Ireland) in center 1, and Innervator^®^ (Fisher-Paykel Health Care, Baxter, Maurepas, France) in center 2. The TOF delivers 4 supramaximal electrical impulses that involve four equally strong twitches of the stimulated muscle. A fade of the twitches appears when the neuromuscular blockade increases [10, 11]. To homogenize practices, both neuromuscular monitors were used in the same way, without acceleromyography: the intensity of the stimulating current was set at 50 mA, and the TOF count was visually assessed. After cleaning the skin, monitoring of the neuromuscular blockade was performed using surface electrodes (Red Dot^®^, 3M Health Care, Neuss, Germany) on the temporal branch of the facial nerve and on the ulnar nerve at the wrist, with assessment of twitches of the orbicularis oculi and adductor pollicis muscles, respectively [20, 21]. The presence or absence of edema was noted before each measurement. Electrodes were changed at least once per day. Monitoring was performed twice a day by one of the investigators. At each assessment, we asked the senior physician in charge, blinded about results of the TOF, to evaluate comprehensively the depth of muscle relaxation as “over-paralyzed,” “well-paralyzed,” or “under-paralyzed.” In both ICUs, the clinical assessment was based on the attentive observation of patient’s movements, ventilator asynchronies, respiratory pressures, and presence of cough or not [22]. Measurements of the TOF on the ulnar nerve were performed arbitrary 4 h after the end of the infusion of the NMBAs, that is beyond five elimination half-lives, to record residual paralysis, and patients with TOF < 4 were reassessed at 8 h.

### Data collection

The following variables were recorded: age, gender, body mass index, Simplified Acute Physiologic Score II [23], and Sequential Organ Failure Assessment the day of NMBA initiation [24]. In addition, comorbidities, which could affect the pharmacokinetics of NMBAs or risk of ICU-AW, were noted: diabetes mellitus, cirrhosis, renal disease, corticosteroid treatment [1, 25]. We also noted the reasons for ICU admission and neuromuscular blockade, total doses, and duration of hypnotics and opioids.

All ARDS patients received protective ventilation and were ventilated as follows: assist-control mode, initial tidal volume targeted at 6 mL per kilogram of predicted body weight, positive end-expiratory pressure (PEEP) level was selected from the PEEP-FiO2 table proposed by the ARDS network [26], and end-inspiratory pressure was measured to be kept below 30 cm of water. Therefore, we recorded the major components of these parameters at the time of each measurement, i.e., the plateau pressure, the calculated driving pressure, and the ratio of the partial pressure of arterial oxygen (PaO2) to the fraction of inspired oxygen (FiO2). Of the treatments received, we noted the duration of catecholamines and use of renal replacement therapy. Duration of MV, duration of ICU and hospital stay, ICU and hospital mortality were finally recorded.

Muscle strength was evaluated with the use of the Medical Research Council (MRC) scale, 7 days after awakening the patient, as previously described [27]. This scale assesses six muscle groups, with a score for each group from 0 (paralysis) to 5 (normal strength). ICU-AW was defined by an overall score of less than 48/60 [27].

### Definitions and endpoints

Based on 2002 and 2008 recommendations [2, 4], three levels of neuromuscular blockade were determined: “over-paralyzed” defined by a TOF = 0/4, “well-paralyzed” defined by a TOF = 1–2/4, and “under-paralyzed” defined by a TOF = 3–4/4. At each measurement, the patient was classified in one of the three categories for the facial and the ulnar sites. Regarding clinical assessment, physicians were asked to judge the adequacy of the muscle relaxation, taking into account the clinical context. Consequently in some cases depth of neuromuscular blockade could be considered as excessive by some physicians.

The primary endpoint was to compare the proportions of patients classified as “over-paralyzed,” “well-paralyzed,” or “under-paralyzed” between the ulnar and facial TOF and physicians’ clinical assessment and to assess agreements between measurements. The secondary endpoints included an analysis of trends in the TOF values over time and comparisons of recorded plateau pressures, calculated driving pressures, and PaO2/FiO2 ratios according to the facial TOF levels in the subgroup of ARDS patients. The third endpoint was to determine whether results for TOF measurements were associated with ICU-AW.

### Statistical analysis

Continuous variables were expressed as medians and interquartile ranges and were compared using a nonparametric Mann–Whitney *U* test or a Kruskal–Wallis test. Categorical variables were expressed as numbers and percentages and were compared using a Chi-square test or a Fisher exact test and a Chi-square test for trends when required. The tests were two-sided, and we considered a *p* value of less than 0.05 to be statistically significant. Treating the levels of neuromuscular blockade as categorical, a kappa statistic was calculated to analyze the agreement between the facial TOF measurements, the ulnar TOF measurements, and the clinical assessments. Statistical analyses were performed using MedCalc, version 11.3.3.0 (MedCalc Software, Mariakerke, Belgium).

## Results

### Patients’ characteristics

**Fig. 1:**
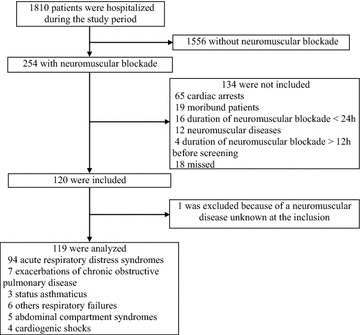
Flowchart of patients

During the study period, 254 patients who received NMBAs were screened for the study. Of these patients, 134 could not be included. One of the 120 patients included was finally excluded because of a chronic neurological disease unknown at the time of inclusion (Fig. 1). Of the 119 patients included in the study, 97 (82%) patients were hospitalized in center 1 and 22 (18%) in center 2. The main characteristics of the patients are shown and distinguished according to the inclusion center in Table 1. The main reason for the neuromuscular blockade was ARDS. Patients differed significantly between the two centers for main reason for ICU admission and neuromuscular blockade. Of note, all patients in center 1 received cisatracurium and all patients in center 2 received atracurium.

**Table 1:** Patients’ characteristics at the inclusion

Characteristics	All patients *N* = 119	Center 1 (cisatracurium use) *N* = 97	Center 2 (atracurium use) *N* = 22
Age (years)	62 (52–73)	62 (51–72)	63 (52–73)
Male [*n* (%)]	78 (66)	61 (63)	17 (77)
Body mass index (kg/m^2^)	25.5 (23.4–29.6)	25.3 (23.1–29.6)	26.6 (23.7–32.2)
SAPS II (points)	51 (40–62)	50 (40–62)	51 (44–60)
SOFA (points)	9 (6–12)	9 (7–12)	9 (6–12)
Length of stay before ICU (days)	1 (0–4)	1 (0–4)	1 (0–3)
Main reason for ICU admission [*n* (%)]			
Medical	111 (93)	93 (96)	18 (82)*
Surgical	8 (7)	4 (4)	4 (18)
Main reason for neuromuscular blockade [*n* (%)]			
ARDS	94 (79)	78 (80)	16 (73)*
Exacerbations of COPD	7 (6)	5 (5)	2 (9)
Status asthmaticus	3 (3)	3 (3)	0
Other respiratory failures	6 (5)	5 (5)	1 (4.5)
Abdominal compartment syndromes	5 (4)	2 (2)	3 (13.5)
Miscellaneous	4 (3)	4 (4)	0
Main comorbidities [*n* (%)]			
Diabetes	16 (13)	14 (14)	2 (9)
Renal disease	8 (7)	7 (7)	1 (5)
Cirrhosis	16 (13)	14 (14)	2 (9)
Corticosteroid treatment	12 (10)	11 (11)	1 (5)
Sepsis [*n* (%)]	85 (71)	68 (70)	17 (77)
Catecholamines [*n* (%)]	78 (66)	62 (64)	16 (73)
Renal replacement therapy [*n* (%)]	9 (8)	6 (6)	3 (14)

Continuous variables are reported as medians and interquartile ranges (25th–75th percentiles), and categorical variables as numbers and percentages

*SAPS II* Simplified Acute Physiologic Score II, *SOFA* Sequential Organ Failure Assessment, *ICU* intensive care unit, *ARDS* acute respiratory distress syndrome, *COPD* chronic obstructive pulmonary disease

* *p* < 0.05 center 1 versus center 2

For the whole population, the duration of invasive MV was 11 days [7–24]; the length of stay in the ICU was 14 days [8–26]; and the length of stay in the hospital was 27 days [15–56]. Forty-seven (39%) patients died in the ICU, and 6 (5%) additional patients died in the hospital. ICU mortality rates did not differ between centers after comparison (*p* = 0.15).

### Neuromuscular blockade

At inclusion, the NMBA infusion rates were 0.18 mg kg^−1^ h^−1^ [0.16–0.22] for cisatracurium and 0.52 mg kg^−1^ h^−1^ [0.47–0.63] for atracurium. For the whole population, the median duration of the NMBA infusion was 67 h [38–121], without differences between the two centers (64 h [38–124] for center 1 vs. 85 h [40–118] for center 2, *p* = 0.37). At the end of the NMBA infusion, the ulnar TOF was performed in all the assessable patients (*n* = 98). Six (6%) had TOF < 4 at 4 h. Of them, 4 could be reassessed at 8 h, and they all had a TOF of 4/4.

### Comparisons between TOF measurements and clinical assessments

On the whole population 996 TOF facial and ulnar measurements and 996 clinical assessments were performed, 846 groups of measurements in patients receiving cisatracurium (center 1) and 150 groups of measurements in patients receiving atracurium (center 2).

**Fig. 2:**
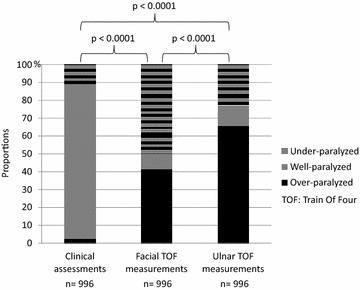
Comparison of results obtained by the train of four and the clinical assessment of neuromuscular blockade recorded over the entire study period

On the whole population proportions of patients classified as “over-paralyzed,” “well-paralyzed,” or “under-paralyzed” differed significantly when results for clinical assessments were compared with results obtained with facial and ulnar TOF measurements (Fig. 2). Based on clinical assessment patients were considered to have adequate muscle relaxation nearly 9 out of 10 times while only 1 patient out of 10 was considered well-paralyzed according to TOF measurements. Statistical results were similar whether comparisons were performed on the whole population or in subgroups of patients receiving cisatracurium or atracurium (Additional file 1: Table S1).

When compared, results obtained with facial and ulnar TOF measurements differed significantly. Compared to results obtained at ulnar site, patients were classified twice as often under-paralyzed based on results obtained at facial site.

Of note, edema did not modify TOF values since there was no difference in patients with edema compared to patients without edema (data not shown).

### Agreement between TOF measurements and clinical assessments

Based on kappa statistic, we found similar levels of agreement for comparisons between TOF measurements and clinical assessments in center 1 and center 2 (Additional file 2: Tables S2a–S2i). When the whole population was considered, the number of observed agreements was 190 (19.08%) (*κ* = 0.06) between clinical assessments and facial TOF measurements and of 173 (17.37%) (*κ* = 0.04) between clinical assessments and ulnar TOF measurements. The level of agreement was slightly higher for comparison between facial und ulnar measurements since the number of observed agreements was of 625 (62.75%) (*κ* = 0.45).

### Subgroup analysis on ARDS patients

**Fig. 3:**
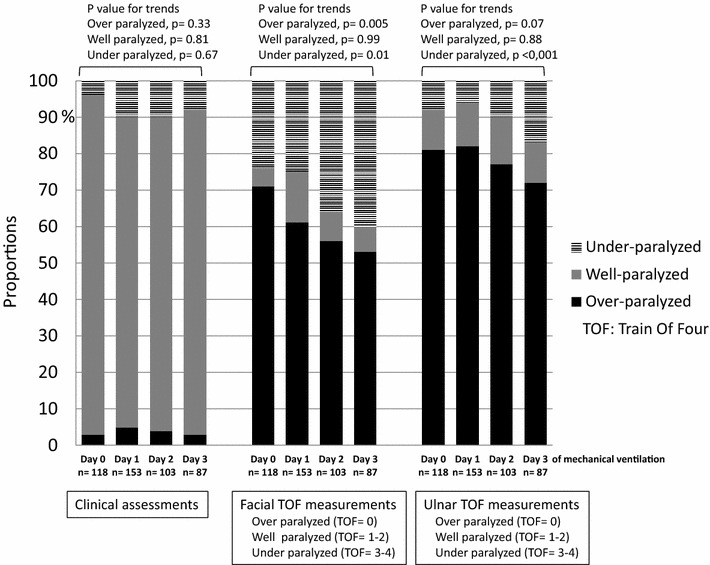
Clinical, facial, and ulnar train-of-four (TOF) measurements recorded on the first 4 days of mechanical ventilation from the diagnosis of ARDS

We analyzed data recorded on the first 4 days of MV for time analysis. Results for clinical assessments did not vary significantly with time. Based on ulnar and facial TOF measurements, from day 0 to day 3 of MV the proportion of patients considered “well-paralyzed” did not vary significantly while the proportion of patients “under-paralyzed” increased significantly. The proportion of patients “over-paralyzed” decreased significantly with time according to facial TOF measurements and tended to decrease significantly according to ulnar measurements (Fig. 3). Similar results were found whether patients received cisatracurium or atracurium (Additional file 3: Table S3). When PaO2/FiO2 ratios, plateau pressures, and calculated driving pressures were compared daily between the three different levels of neuromuscular blockade defined by the facial measurements of the TOF on the first 4 days of MV, no significant difference was found (Table 2). The daily dose of cisatracurium used in center 1 increased from 150 mg (90–240, day 0) to 300 mg (166–330, day 3) (*p* < 0.01 after comparison), and the daily dose of atracurium used in center 2 increased from 219 mg (153–235, day 0) to 362 mg (217–454, day 3) (*p* = 0.03 after comparison).

**Table 2:** PaO2/FiO2 ratios, plateau pressure, and calculated driving pressure recorded on the first 4 days of mechanical ventilation (MV) from the diagnosis of ARDS, distinguished according to results for facial train of four (TOF), and compared day by day

Day of MV	Day 0		Day 1		Day 2		Day 3	
	First	Second	First	Second	First	Second	First	Second
Measurements (*n*)	80	38	88	65	66	37	51	36
PaO2/FiO2 (mmHg), median (IQR)*								
Facial TOF = 0	115 (82–154)	170 (100–209)	148 (116–231)	171 (143–192)	178 (128–212)	151 (105–196)	160 (118–201)	136 (114–183)
Facial TOF = 1–2	111 (93–206)	160 (107–220)	203 (121–269)	142 (121–178)	198 (154–228)	–	183 (146–213)	121 (113–130)
Facial TOF = 3–4	98 (77–148)	168 (137–246)	159 (128–198)	195 (162–239)	185 (140–232)	220 (210–240)	187 (130–211)	154 (140–184)
Plateau pressure, cmH2O, median (IQR)*								
Facial TOF = 0	24 (19–26)	22 (20–25)	23 (19–25)	22 (20–24)	22 (20–24)	24 (20–25)	23 (20–25)	23 (22–26)
Facial TOF = 1–2	27 (23–27)	23 (23–24)	21 (17–23)	21 (19–27)	20 (18–20)	–	24 (21–26)	21 (17–25)
Facial TOF = 3–4	22 (20–27)	21 (20–22)	22 (20–24)	22 (21–24)	22 (20–24)	22 (20–24)	22 (19–24)	23 (20–25)
Driving pressure, cmH2O, median (IQR)*								
Facial TOF = 0	11 (9.5–18)	11 (8.5–14)	11 (9–13)	10 (9–12)	11 (9–13)	12 (10–14)	12 (11–17)	13 (11–17)
Facial TOF = 1–2	12 (12–14)	11 (10–12)	9 (9–11.5)	10 (8–15)	9.5 (8–11.5)	–	11.5 (10–14)	10 (9–11)
Facial TOF = 3–4	12 (10–16)	10.5 (8.5–12)	11 (10–14)	11 (9.5–13)	11 (9–13)	11 (8.5–12.5)	10 (9.5–13.5)	12 (9.5–15)

*IQR* interquartile ranges

* No significant difference for the daily first and second measurements when compared between results obtained for facial TOF = 0, facial TOF = 1–2, and facial TOF = 3–4

### ICU-acquired weakness 7 days after awakening the patient

The MRC score could be assessed in 47 patients who were able to follow commands 7 days after awakening. ICU-AW was diagnosed in 14 patients (30%). After comparisons between patients with and without ICU-AW, proportions of patients with liver cirrhosis [0 (0%) vs. 5 (15%), respectively, *p* = 0.32], diabetes mellitus [1 (14%) vs. 2 (6%), respectively, *p* = 0.45], renal disease [0 (0%) vs. 3 (9%), respectively, *p* = 0.42] and who received concomitant administration of steroids during the ICU stay [9(64%) vs. 15 (33%), respectively, *p* = 0.23] did not differ significantly. The other risk factors for ICU-AW assessed in the study did not differ significantly between patients with and without ICU-AW (Additional file 4: Table S4). Proportion of ulnar and facial TOF = 0 during treatment with NMBAs was significantly higher in patients with ICU-AW than in patient without ICU-AW, although it was not significant for total doses and duration of cisatracurium and atracurium (Table 3).

**Table 3:** Neuromuscular blockade, train-of-four (TOF) measurements, and intensive care unit-acquired weakness (ICU-AW) 7 days after awaking

	ICU-AW = Yes	ICU-AW = No	*p* value
Whole population	*N* = 14	*N* = 33	
Measures of facial TOF = 0/all measures of facial TOF	75/147 (51%)	97/243 (40%)	0.03
Measures of ulnar TOF = 0/all measures of ulnar TOF	111/147 (76%)	151/243 (62%)	0.006
Center 1	*N* = 12	*N* = 29	
Cisatracurium total dose (mg)	1482 (635–3257)	998 (591–1470)	0.25
Cisatracurium duration (h)	89 (42–210)	65 (35–110)	0.28
Center 2	*N* = 2	*N* = 4	
Atracurium total dose (mg)	1056 (1092–1320)	472 (418–749)	0.06
Atracurium duration (h)	190 (186–195)	93 (76–105)	0.07

Continuous variables are reported as medians and interquartile ranges (25th–75th percentiles)

*ICU*-*AW* intensive care unit-acquired weakness, *NMBAs* neuromuscular blocking agents, *TOF* train of four

## Discussion

To our knowledge, here we report the largest prospective cohort of critically ill patients paralyzed with the recommended doses of benzylisoquinoliniums and assessed for neuromuscular blockade by both a clinical evaluation and ulnar and facial TOF measurements. As expected we found that the evaluation of neuromuscular blockade differed vastly between the TOF and clinical assessment but also that TOF counts differed significantly between evaluation of facial and hand muscles. Furthermore, we found that the proportion of TOF = 0 measurements was significantly higher in patients with ICU-AW than in patients with no ICU-AW. An important result is that findings did not differ whether patients received cisatracurium or atracurium.

As it is already known in the operating room setting [10, 11], our study shows that an overall clinical assessment of the depth of a neuromuscular blockade is not correlated with the TOF count in critically ill patients. This finding has already been described in three previous studies, where the clinical assessment was performed by nurses and not by physicians, and two of these studies took place in pediatric ICUs [15–17]. Our results highlight the major discrepancy between clinical assessment and measures obtained after PNS but also between ulnar and facial TOF measurements.

A major finding of this study was that the objective of TOF count of 1 or 2 was obtained in only less than 10% of the measurements when patients are monitored only according to clinical assessment, even though the NMBA infusion rates were in accordance with the recommendations [2, 4]. Few studies supported the recommendations of monitoring the TOF in the ICU. Rudis et al. [8] compared in one group of patients paralyzed with an aminosteroid managed with a clinical assessment and one group managed with a TOF for an objective of 1/4. These authors observed a decreased amount of infused NMBAs and a faster recovery of muscle paralysis and spontaneous ventilation in the TOF group. However, in studies using benzylisoquinoliniums, there was no advantage for amount of infused NMBAs or residual weakness with a TOF compared to a clinical assessment [22, 28]. It is noteworthy that in the 2016 American recommendations, the only indication for NMBAs is ARDS with a ratio PaO2/FiO2 less than 150, and that no dosage and no objective of TOF are advocated [5].

A favorable impact of a neuromuscular blockade on the 90-day survival for severe ARDS patients was demonstrated in a well-designed study [3] with higher doses than recommended (i.e., 37.5 mg/h of cisatracurium), without monitoring the TOF. These doses were based on two previous studies from the same group with a goal of zero twitch for everyone during 48 h [29, 30]. This favorable effect of the NMBAs on ARDS may be due to an improvement in adjustment of tidal volume and plateau pressure with a reduction of asynchronies and ventilator-induced lung injuries, to an improvement of oxygenation, and finally to an anti-inflammatory effect [3, 29–31]. In one study, an objective of a TOF at 0/4 was compared to an objective at 2/4 [32]. As in our study, the plateau pressure and ratio PaO2/FiO2 did not differ between the TOF values. However, we did not assess asynchronies and we did not measure levels of cytokines, and thus, we cannot exclude that a deeper level of neuromuscular blockade is required to prevent lesions induced by bio-traumatism. Repeated measurements suggest also that higher than recommended and increasing doses of NMBAs during the first days of MV are required to obtain no facial TOF response and consequently total paralysis of the diaphragmatic and accessory muscles [3, 29, 30].

The proportion of TOF 3 or 4 increased significantly with time while patients receive the recommended doses of NMBAs. This has already been attributed to an up-regulation of acetylcholine receptors, although some authors did not find the same results [13, 22]. The pharmacokinetics of NMBAs in critically ill patients can be affected by many conditions, such as sepsis and/or shock [12]. In addition, it has been shown that ICU patients are less sensitive to neuromuscular blockade than patients in the operating room [14].

The link between NMBAs and ICU-AW, especially with benzylisoquinoliniums, is debated [1, 25, 27, 33, 34]. We diagnosed ICU-AW in 30% of our patients able to perform the MRC score on day 7 after their awakening, which is in accordance with the literature [25, 27]. The percentages of the ulnar and facial TOF = 0 measurements were higher in the cases of ICU-AW, while total doses and duration of NMBAs were not associated with a higher risk of ICU-AW. Further studies are required to determine whether that aiming for an objective of TOF count of 1 or 2 rather than zero could reduce amounts of infused NMBAs, thus lowering the risk of ICU-AW. In addition, there are many risk factors for ICU-AW which could be confounders [25, 27] and the present study was underpowered to assess most of them.

The results showing that TOF = 0 is more frequent at the ulnar site is not surprising since the orbicularis oculi muscle is known to be less sensitive to NMBAs than the adductor pollicis muscle. We monitored the TOF on the facial and the ulnar nerves because the facial site is advised in the French recommendations, but not in the recommendations from the Society of Critical Care Medicine [2, 4]. The orbicularis oculi muscle better assesses the depth of relaxation of the diaphragm, which is often the goal of a neuromuscular blockade in the ICU, especially in cases of ARDS [29, 30]. Although our study was not designed to, it seems more appropriate to monitor the facial TOF in ARDS patients to provide the adequate dose of NMBAs, at least during the first 48 h of MV in order to totally paralyze the diaphragmatic muscle as suggested by Papazian et al. [3]. Nevertheless, it is currently impossible to determine the aim of TOF to achieve in ARDS patients because in the only study [3] that shown a benefit in the use of NMBAs, no monitoring of TOF was done. Of note, the two centers did not use the same peripheral nerve stimulator and we believe that differences in NMBAs and TOF monitors between centers increase the external validity of the study.

Our study has some limitations. We included mainly medical patients so our results may not be generalized to all ICUs. We did not use an automated TOF measurement. More measurements would have provided a more accurate assessment, although the best frequency for measurement is not specified in the literature [1, 2, 4]. Ideally, the intensity of the stimulating current has to be tested for each patient before an NMBA infusion, which is difficult in real practice. Thus, we chose an intensity of the stimulating current of 50 mA for all patients, which was in agreement with some authors [20, 21]. Although we restrained ourselves to follow the advice published in the literature, the measurement of the TOF can be impaired by technical pitfalls such as incorrect positioning of electrodes [20, 21, 35]. The clinical evaluation of a neuromuscular blockade by physicians was not recorded in a standardized form, and we did not find models in the literature. The depth of sedation may have influenced the clinical evaluation of the neuromuscular blockade. Asynchronies and spontaneous breathing efforts were not precisely and extensively recorded in ARDS patients. Finally, we did not assess results of TOF on extubation times mainly because weaning process differed slightly between the two centers.

In conclusion, our study shows that there is a huge discrepancy between the clinical assessment and the TOF measurements in critically ill patients, that TOF count of 1 or 2 is a goal rarely achieved at usual doses of NMBAs, but also that respiratory objectives for plateau pressure and oxygenation can be obtained in ARDS patients without TOF monitoring. Our results do not suggest that the need for monitoring NMBAs could itself be questioned but rather that site for nerve stimulation and objective for TOF count could differ according to critically ill patients, reasons for neuromuscular blockade, and courses of the diseases. Nevertheless, more studies are needed to establish the best target of the TOF count to provide necessary and sufficient muscle relaxation and avoid residual muscular weakness.

## Additional files

**Additional file 1.** Comparisons of results obtained by the train of four and the clinical assessment of neuromuscular blockade recorded over the entire study period.

**Additional file 2.** Results for agreements between clinical assessment of neuromuscular blockade with the train of four on the facial and ulnar nerves on whole population, in center 1 and in center 2.

**Additional file 3.** Clinical, facial and ulnar train of four measurements recorded on the first 4 days on mechanical ventilation from the diagnosis of acute respiratory distress syndrome.

**Additional file 4.** Factors associated with the diagnosis of intensive care unit-acquired weakness seven days after awaking the patient.

## Abbreviations

ARDS: acute respiratory distress syndrome
ICU-AW: intensive care unit-acquired weakness
MRC: Muscular Research Council
MV: mechanical ventilation
NMBAs: neuromuscular blocking agents
PaO2/FiO2: ratio of the partial pressure of arterial oxygen (PaO2) to the fraction of inspired oxygen (FiO2)
PEEP: positive end-expiratory pressure
PNS: peripheral nerve stimulation
TOF: train of four
