# Supplementary material for: Clinical assessment and train-of-four measurements in critically ill patients treated with recommended doses of cisatracurium or atracurium for neuromuscular blockade: a prospective descriptive study
Source: Ann Intensive Care. 2017 Jan 19;7:10. doi: 10.1186/s13613-017-0234-0 (PMC5247382; doi:10.1186/s13613-017-0234-0)
Supplement: Supplementary file 1 — Additional file 1. Comparisons of results obtained by the train of four and the clinical assessment of neuromuscular blockade recorded over the entire study period. [file 13613_2017_234_MOESM1_ESM.pdf]

Additional file 1: Table S1.

Table S1: Comparison of results obtained by the train of four and the clinical assessment of neuromuscular blockade recorded over the entire study period.

	TOF measurements		Clinical assessments
	Facial	Ulnar	
**Whole population**	n = 996	n = 996	n = 996
- over-paralyzed (TOF = 0), n (%)	412 (41.4)	653 (65.6) [a]	27 (2.7) [b,c]
- well-paralyzed (TOF = 1-2), n (%)	88 (8.8)	114 (11.4)	859 (86.2)
- under-paralyzed (TOF = 3-4), n (%)	496 (49.8)	229 (23)	110 (11.1)
**Center 1 (Cisatracurium use)**	n = 846	n = 846	n = 846
- over-paralyzed (TOF = 0), n (%)	359 (42.4)	545 (64.5) [a]	14 (1.7) [b,c]
- well-paralyzed (TOF = 1-2), n (%)	78 (9.2)	101 (11.9)	748 (88.4)
- under-paralyzed (TOF = 3-4), n (%)	409 (48.4)	200 (23.6)	84 (9.9)
**Center 2 (Atracurium use)**	n = 150	n = 150	n = 150
- over-paralyzed (TOF = 0), n (%)	53 (33.5)	108 (72) [a]	13 (8.6) [b,c]
- well-paralyzed (TOF = 1-2), n (%)	10 (6.6)	13 (8.6)	111 (74)
- under-paralyzed (TOF = 3-4), n (%)	87(58)	29 (19.4)	26 (17.4)

Variables are reported as numbers and percentages.

TOF: Train Of Four.

[a] facial TOF vs. ulnar TOF, $p < 0.0001$.

[b] facial TOF vs. clinical assessment, $p < 0.0001$.

[c] ulnar TOF vs. clinical assessment, $p < 0.0001$.
