# Supplementary material for: Clinical assessment and train-of-four measurements in critically ill patients treated with recommended doses of cisatracurium or atracurium for neuromuscular blockade: a prospective descriptive study
Source: Ann Intensive Care. 2017 Jan 19;7:10. doi: 10.1186/s13613-017-0234-0 (PMC5247382; doi:10.1186/s13613-017-0234-0)
Supplement: Supplementary file 2 — Additional file 2. Results for agreements between clinical assessment of neuromuscular blockade with the train of four on the facial and ulnar nerves on whole population, in center 1 and in center 2. [file 13613_2017_234_MOESM2_ESM.pdf]

Additional file 2: Results for agreements between clinical assessment of neuromuscular blockade with train of four (TOF) on the facial and ulnar nerves on whole population, in center 1 and in center 2.

Table S2a: Comparison on whole population of the train of four (TOF) on the facial nerve and the clinical assessment of neuromuscular blockade.

	Clinical assessment « over-paralyzed »	Clinical assessment « well-paralyzed »	Clinical assessment « under-paralyzed »	Total
Facial TOF = 0	15	382	15	412
Facial TOF = 1-2	1	84	3	88
Facial TOF = 3-4	11	394	91	496
Total	27	860	109	996

The agreement was very poor between the facial TOF and the clinical assessment (kappa: 0.06; weighted kappa: 0.09).

Table S2b: Comparison in center 1 (Cisatracurium use) patients of the train of four (TOF) on the facial nerve and the clinical assessment of neuromuscular blockade.

	Clinical assessment « over-paralyzed »	Clinical assessment « well-paralyzed »	Clinical assessment « under-paralyzed »	Total
Facial TOF = 0	9	342	10	361
Facial TOF = 1-2	1	74	1	76

Facial TOF = 3-4	4	333	72	412
Total	14	749	83	846

The agreement was very poor between the facial TOF and the clinical assessment (kappa: 0.057; weighted kappa: 0.09).

Table S2c: Comparison in center 2 (atracurium use) patients of the train of four (TOF) on the facial nerve and the clinical assessment of neuromuscular blockade.

	Clinical assessment « over-paralyzed »	Clinical assessment « well-paralyzed »	Clinical assessment « under-paralyzed »	Total
Facial TOF = 0	6	40	5	51
Facial TOF = 1-2	0	10	2	12
Facial TOF = 3-4	7	61	91	19
Total	13	111	26	150

The agreement was very poor between the facial TOF and the clinical assessment (kappa: 0.054; weighted kappa: 0.08).

Table S2d: Comparison on whole population of the train of four (TOF) on the ulnar nerve and the clinical assessment of neuromuscular blockade.

	Clinical assessment « over-paralyzed »	Clinical assessment « well-paralyzed »	Clinical assessment « under-paralyzed »	Total
Ulnar TOF = 0	22	594	37	653
Ulnarl TOF = 1-2	1	96	17	114
Ulnar TOF = 3-4	4	170	55	229
Total	27	860	109	996

The agreement was very poor between the facial TOF and the clinical assessment (kappa: 0.04; weighted kappa: 0.07).

Table S2e: Comparison in center 1 (Cisatracurium use) patients of the train of four (TOF) on the ulnar nerve and the clinical assessment of neuromuscular blockade.

	Clinical assessment « over-paralyzed »	Clinical assessment « well-paralyzed »	Clinical assessment « under-paralyzed »	Total
Ulnar TOF = 0	8	535	21	564
Ulnarl TOF = 1-2	0	88	13	101
Ulnar TOF = 3-4	1	140	40	184
Total	9	763	74	846

The agreement was very poor between the facial TOF and the clinical assessment (kappa: 0.031; weighted kappa: 0.067).

Table S2f: Comparison in center 2 (atracurium use) patients of the train of four (TOF) on the ulnar nerve and the clinical assessment of neuromuscular blockade.

	Clinical assessment « over-paralyzed »	Clinical assessment « well-paralyzed »	Clinical assessment « under-paralyzed »	Total
Facial TOF = 0	14	59	16	89
Facial TOF = 1-2	1	8	4	13
Facial TOF = 3-4	3	30	15	48
Total	18	97	35	150

The agreement was very poor between the facial TOF and the clinical assessment (kappa: 0.056; weighted kappa: 0.097).

Table S2g: Comparison on whole population of the train of four (TOF) recorded on the facial and ulnar nerves.

	Facial TOF = 0	Facial TOF = 1-2	Facial TOF = 3-4	Total
Ulnar TOF = 0	399	68	186	653
Ulnar TOF = 1-2	8	11	95	114
Ulnar TOF = 3-4	5	9	215	229
Total	412	88	496	996

The agreement was very moderate between the ulnar and facial TOF (kappa: 0.38; weighted kappa: 0.45).

Table S2h: Comparison in center 1 (Cisatracurium use) patients of the train of four (TOF) recorded on the facial and ulnar nerves.

.

	Facial TOF = 0	Facial TOF = 1-2	Facial TOF = 3-4	Total
Ulnar TOF = 0	351	59	139	548
Ulnar TOF = 1-2	7	8	82	97
Ulnar TOF = 3-4	4	9	188	201
Total	362	76	411	846

The agreement was very moderate between the ulnar and facial TOF (kappa: 0.41; weighted kappa: 0.48).

Table S2i: Comparison in center 2 (atracurium use) patients of the train of four (TOF) recorded on the facial and ulnar nerves.

	Facial TOF = 0	Facial TOF = 1-2	Facial TOF = 3-4	Total
Ulnar l TOF = 0	48	9	48	105
Ulnar TOF = 1-2	1	3	13	17
Facial TOF = 3-4	1	0	27	28
Total	50	12	88	150
