# Supplementary material for: Clinical assessment and train-of-four measurements in critically ill patients treated with recommended doses of cisatracurium or atracurium for neuromuscular blockade: a prospective descriptive study
Source: Ann Intensive Care. 2017 Jan 19;7:10. doi: 10.1186/s13613-017-0234-0 (PMC5247382; doi:10.1186/s13613-017-0234-0)
Supplement: Supplementary file 3 — Additional file 3. Clinical, facial and ulnar train of four measurements recorded on the first 4 days on mechanical ventilation from the diagnosis of acute respiratory distress syndrome. [file 13613_2017_234_MOESM3_ESM.pdf]

Additional file 3: Table S3

Table S2: Clinical, facial and ulnar train of four (TOF) measurements recorded on the first 4 days of mechanical ventilation (MV) from the diagnosis of acute respiratory distress syndrome. .

	Whole population				P value	Center 1 (Cisatracurium use)				P value	Center 2 (Atracurium use)				P value
Day of MV	0	1	2	3		0	1	2	3		0	1	2	3	
Measurements, n	118	153	103	87		98	127	87	72		20	26	16	15	
Clinical assessments, n (%)															
Over paralyzed	5 (3)	7 (5)	4 (4)	2 (3)	0.33	4 (4)	3 (2)	3 (3)	1 (1)	0.41	1 (10)	4 (19)	1 (7)	1 (13)	0.88
Well paralyzed	109 (93)	130 (85)	89 (86)	78 (89)	0.81	91 (92)	114 (91)	78 (90)	67 (94)	0.92	18 (90)	16 (61)	11 (68)	11 (67)	0.31
Under paralyzed	4 (4)	16 (14)	10 (10)	7 (8)	0.67	3 (3)	9 (7)	6 (7)	4 (5)	0.49	1 (10)	5 (20)	4 (25)	3 (20)	0.19
Facial TOF measurements, n (%)															
Over paralyzed (TOF= O)	84 (71)	94 (61)	58 (56)	46 (53)	0.005	75 (77)	84 (66)	54 (62)	41 (57)	0.006	9 (45)	10 (38)	4 (25)	5 (33)	0.30
Well paralyzed (TOF= 1-2)	6 (5)	21 (14)	8 (8)	6 (7)	0.99	3 (3)	18 (14)	6 (7)	6 (8)	0.54	3 (5)	3 (12)	2 (13)	0	0.19

Under paralyzed (TOF= 3-4)	28 (24)	38 (25)	37 (36)	35 (40)	0.01	20 (20)	25 (20)	27 (31)	25 (35)	0.009	8 (40)	13 (50)	10 (62)	10 (67)	0.08
Ulnar TOF measurements, n (%)															
Over paralyzed (TOF= O)	96 (81)	126 (82)	79 (77)	63 (72)	0.07	79 (80)	104 (82)	68 (78)	52 (72)	0.19	17 (85)	22 (85)	11 (69)	11 (73)	0.22
Well paralyzed (TOF= 1-2)	13 (11)	19 (12)	13 (13)	10 (11)	0.88	11 (11)	17 (13)	9 (10)	8 (11)	0.82	2 (10)	2 (8)	4 (25)	2 (13)	0.40
Under paralyzed (TOF= 3-4)	9 (8)	8 (6)	11 (10)	14 (17)	<0.001	8 (9)	6 (5)	8 (12)	12 (16)	0.04	1 (5)	2 (7)	1 (6)	2 (13)	0.43
