# Supplementary material for: Clinical assessment and train-of-four measurements in critically ill patients treated with recommended doses of cisatracurium or atracurium for neuromuscular blockade: a prospective descriptive study
Source: Ann Intensive Care. 2017 Jan 19;7:10. doi: 10.1186/s13613-017-0234-0 (PMC5247382; doi:10.1186/s13613-017-0234-0)
Supplement: Supplementary file 4 — Additional file 4. Factors associated with the diagnosis of intensive care unit-acquired weakness seven days after awaking the patient. [file 13613_2017_234_MOESM4_ESM.pdf]

Additional File 4

Table S4: Factors associated with the diagnosis of intensive care unit-acquired weakness (ICU-AW) seven days after awakening the patient.

	ICU-AW = YES<br>n = 14	ICU-AW = NO<br>n = 33	p
Age, years	67 (50-73)	59 (48-67)	0.33
Male, n (%)	9 (64)	22 (67)	0.99
Body mass index, kg/m²	26.1 (23.3-28.5)	25.9 (23.1-31.3)	0.66
SAPS II, points	51 (48-69)	46 (34-55)	0.07
SOFA, points	9 (6-12)	8 (6-11)	0.56
Sepsis at inclusion, n (%)	9 (64)	21 (64)	0.99
Duration of sedation, hours	266 (161-391)	204 (104-324)	0.30
Total dose of midazolam, mg	1440 (628-2262)	872 (554-1683)	0.40
Total dose of morphinic, mg	1425 (576-2974)	1251 (591-1977)	0.69
Duration of catecholamines, days	6 (3-9)	4 (3-7)	0.29
Total dose of corticosteroids administered concomitantly to NMBAs , mg	1350 (750-2825)	700 (228-1250)	0.08
Mean glycaemia, mmol/l	8.3 (7.8-9.1)	8 (7.6-8.8)	0.46
Duration of insulin, days	13 (7-19)	8 (3-15)	0.12
Total dose of insulin, UI	350 (180-792)	151 (55-419)	0.11
Use of renal replacement therapy	7 (50)	6 (18)	0.06

Continuous variables are reported as medians and interquartile ranges (25th-75th percentiles), and categorical variables as numbers and percentages.

ICU-AW: intensive care unit-acquired weakness; SAPS II: Simplified Acute Physiologic Score II; SOFA: Sequential Organ Failure Assessment; NMBA: Neuromuscular blocking agents.
